# The impact of semi-automatic versus manually adjusted assessment of global longitudinal strain in post-myocardial infarction patients

**DOI:** 10.1007/s10554-020-01826-4

**Published:** 2020-03-31

**Authors:** Jan Erik Otterstad, Ingvild Billehaug Norum, Vidar Ruddox, Bjørn Bendz, Kristina H. Haugaa, Thor Edvardsen

**Affiliations:** 1 Department of Medicine, Hospital of Vestfold, Tonsberg, Norway; 2 Department of Cardiology, Oslo University Hospital, Rikshospitalet, and University of Oslo, Sognsvannsveien 20, 0372 Oslo, Norway

**Keywords:** Global longitudinal strain, Region of interest, Post-myocardial infarction

## Abstract

There are unresolved questions related to the proper use of editing the region of interest (ROI) for measurements of global longitudinal strain (GLS). The purpose of the present study was to compare the semi-automatic default GLS value by the vendor’s software with manually adjusted GLS and test the impact on GLS measures with different ROI widths. We selected 25 patients post myocardial infarction treated with PCI who had excellent echocardiographic recordings after 2–5 days and 3 months. The different GLS values were assessed from these 50 analyses in three steps. The semi-automatically GLS by default ROIs was compared with manually adjusted ROIs widths selected by an expert and then with manual adjustments, but with fixed ROIs being narrow, medium and wide. Their mean age was 64 (± 12) years, 52% had ST elevation MI and mean LVEF was 52 (± 4)%. Mean default GLS was − 15.3 (± 2.5)% with the widest ROI level selected semi-automatically in 78% of all widths. The mean expert GLS with manually adjusted ROI was − 14.7 (± 2.4)%, and the medium ROI level was selected by the expert in 85% of all examinations. The mean adjusted GLS, but with fixed ROIs widths was − 15.0 (± 2.5%)% with narrow ROI, − 14.7 (± 2.6)% with medium and − 13.5 (± 2.3)% with wide ROI width (p < 0.001 vs. default GLS). The Intra Class Coefficient Correlation between default and manually adjusted expert GLS was 0.93 (p < 0.001). The difference between the default and the manually adjusted expert GLS was neglectable. These findings may represent a simplification of the assessment of GLS that might increase its use in clinical practice. The GLS measurements with a fixed wide ROIs were significantly different from the expert measurements and indicate that a wide ROI should be avoided.

**Electronic supplementary material:**

The online version of this article (10.1007/s10554-020-01826-4) contains supplementary material, which is available to authorized users.

## Introduction

Recent recommendation and guideline papers encourage clinicians to include global longitudinal strain (GLS) by speckle tracking measurements more in routine clinical practice [1–3]. There are, however, reasons to believe that the medical society should be careful about interpretations of regional or segmental analyses [4]. In recent years, GLS has been proven to be a stronger predictor than left ventricular ejection fraction (LVEF) of all-cause mortality and a composite of cardiac death, heart failure hospitalization and malignant arrhythmias across several studies on various cardiac diseases [4–9]. It has become increasingly clear that LVEF provides no additional prognostic information in patients with preserved or mid-range LVEF [1]. The recent ESC guideline in chronic coronary syndromes recommends GLS in patients with EF > 35% for risk stratification [2]. Arguments opposing the use of GLS have been vendor differences, extra time use and uncertainty whether using fixed default) GLS versus performing manual adjustments of location and width of the region of interest (ROI). Issues with extra time consumption has been minimized by providing fixed default GLS measurements assessed from 3 apical views by all vendors, and the extra time used for GLS analyses is now approximately 1–4 min.

A practical guidance in GLS assessment focused on optimal quality image with caution on ROI placement, especially at the annulus and in the apex [10]. The authors also advised that the ROI width “should not be too wide or narrow”, but a more accurate advice is lacking and required. The purpose of the present study was therefore to compare fixed default GLS settings with manually adjusted measurements of ROI location and width.

Our aims were therefore to test if (1) the default GLS settings would deviate significantly from a reference value based on manually adjusted ROI by an expert, (2) a manually chosen ROI width of narrow, medium or wide would influence significantly on GLS values. We included a study population of PCI treated post-MI patients where GLS measurements have shown additional prognostication beyond that of LVEF.

## Patients and methods

The present study is a part of the ongoing Vestfold Strain Event Study (VEST) where post infarction patients treated with primary (STEMI) or early (NSTEMI) PCI have been included in the period April 2016–December 2018 [11].

In brief, consecutive and stabilized patients were transferred from a tertiary invasive center (Oslo University Hospital, Rikshospitalet) after PCI treatment for acute MI. Angiographic assessment of coronary artery stenosis and PCI was performed according to current guidelines [12]. We excluded patients with poor echocardiographic images according to the performing investigators, atrial fibrillation and/or a history of overt heart failure during their index acute MI (Killip class ≥ 2). All were reexamined after 3 months.

The VEST study has been approved by the Regional ethics Committee of Health Region South-East, Norway (2015/2359).

### Echocardiographic examinations

All studies were performed with the same Vivid E9 machine from Vingmed GE Ultrasound, Horten, Norway at baseline and at 3 months follow up. In order to optimize the quality of recordings only two experienced operators (VR and JEO) performed the echocardiographic examinations. We randomly chose 50 echocardiographic examinations obtained from 25 patients without any cardiac events during the 3 months following their acute MI.

#### LV strain measurements

Three to four consecutive heartbeats were recorded from each of the three apical views. End of systole was defined as aortic valve closure registered by continuous Doppler. Adjustment of default GLS was performed according the guidance of Negishi et al. whenever found appropriate by the actual investigator [10]. We excluded images with suboptimal tracking of the endocardium in more than one segment in one single view or if frame rate was below 50 Hz.

### ROI information from GLS assessment

All GLS values had ROI levels prospectively recorded from each of the three apical views for both default and manually adjusted GLS. The adjustments of ROI location when using fixed ROI levels narrow, medium and wide were identical to those applied for manually adjusted GLS where the expert selected optimal ROI widths in each view. During manual adjustments, we chose to avoid the ROI level extra wide due to possible errors related to inclusion of the epicardium and being outside the sector, which inevitably might cause falsely low values. GLS values presented including the ROI level extra wide in default assessments were left unadjusted in order to obtain a representative evaluation of the default system.

### Principles of manual adjustments of ROI

**Fig. 1:**
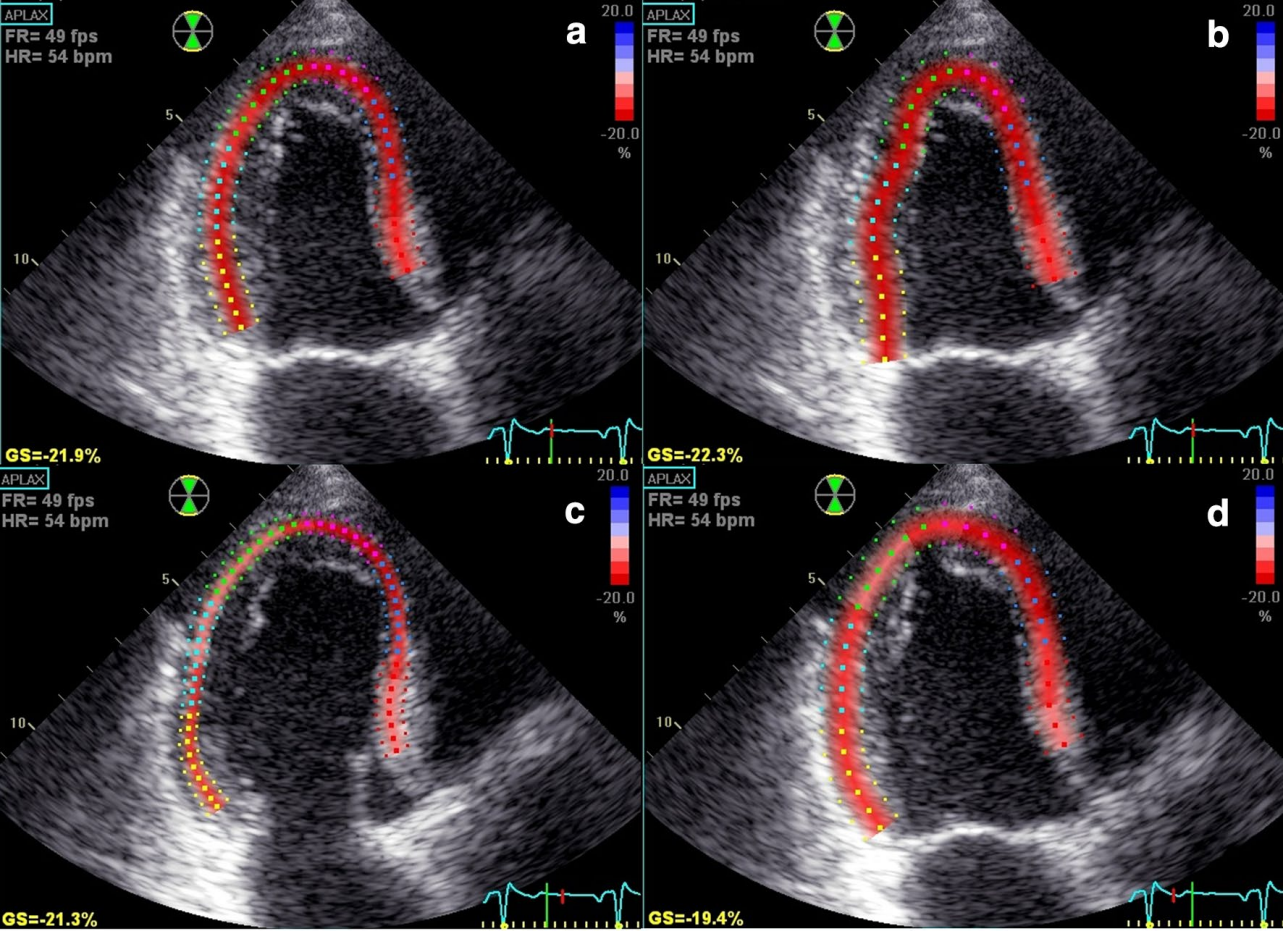
The impact of different ROI adjustments on the GLS values from the apical long chamber view in a 75 years old male with NSTEMI. **a** Manually adjusted expert GLS with ROI level medium selected for the reference value by the expert. GLS = − 21.9%. **b** Default GLS presented with selected ROI level wide by the software. GLS = − 22.3%. **c** Manually adjusted GLS with fixed ROI level narrow. GLS = − 21.3%. **d** Manually adjusted GLS with fixed ROI level wide. GLS = 19.4%

ROI was carefully placed at the level of the mitral cusp insertion while the aortic root was avoided. The inner layer was placed along the identification of the LV endocardium, as applied in LV volume measurements by the Simpson’s rule, cutting through papillary muscles and with inclusion of the apical endocardium. In order to avoid bias, the reference value by manually adjusted approach was measured before assessing the default semi-automated GLS value. Consecutive measurements were then made with different ROI widths, wide, medium and narrow for all three apical views. The different varieties of GLS measurements according to the ROI in this study are exemplified in the long axis views in a patient treated for NSTEMI (Fig. 1).

#### Categories of GLS measurements

1. Default GLS with ROIs as presented by the semi-automatic system without any attempts of adjustments (150 measurements).
2. The expert GLS value with manually adjusted assessment carefully followed the principles for optimal ROI location as stated above. The selection of the ROI did not exceed the echocardiographic frame or included the epicardium. Selection options were narrow, medium or wide (150 measurements).
3. Adjusted GLS by the same expert, but with a fixed narrow, medium and wide ROI levels (450 measurements).

### Conventional echocardiography

#### Apical views

4-chamber and long axis views were used for measurements of LVEF volumes with the biplane Simpson method, and left atrial maximal volume by the biplane area-length method.

Right ventricular areas and area fraction were derived by the single plane area-length method from the 4-chamber view. Pulsed and tissue Doppler were applied for E/e′ ratio, using the average of septal and lateral e′.

### Statistical analysis

Power calculations estimated the required sample size to be n = 49 to detect a 10% difference between default and manually adjusted GLS values with different ROI levels, and with a mean GLS of − 14.9 (± 2.6)% in the 159 interim study population (9). A significance level of 0.05 was used.

Data is either presented as mean ± SD or median (IQR) whenever appropriate. Intergroup differences were evaluated by a paired T-test for normally distributed data Normality was tested with the Shapiro–Wilk whenever visual assessment of the distribution was dubious. For evaluation of the manually adjusted reference GLS versus default GLS, Pearson’s linear correlation was used. In addition, the reliability of the adjusted reference versus default value was assessed by using Intraclass Correlation Coefficient (ICC).

## Results

The baseline characteristics of the 25 patients who were examined twice are provided in Table 1. The patients were dominantly men (68%) in their mid-sixties and 1/5 have suffered from an earlier myocardial infarct. Baseline echocardiography was performed on average 2 (± 0.5) days following PCI, and repeated in the same patients after 3 (± 0.3) months (Table 2). The expert chose the medium width in the majority of all analyses, while the default measures by the software preferred a wide width (Table 3). The difference in ROI preference between the default system and the expert did not, however, influence significantly on the GLS results. Likewise, the manually adjusted GLS levels obtained with fixed ROI of narrow or medium widths were equal to the manually adjusted measures by an expert and the default GLS (Table 4). The only manually adjusted GLS value that was significantly different compared to the default value (− 15.3%) was obtained when a fixed ROI of wide level was used (− 13.5%, Table 4). The absolute difference of − 1.8% (p < 0.001) between these two approaches corresponded to a 12% relative difference compared to the manually adjusted expert GLS values.

**Table 1:** Baseline characteristics of 25 patients included and examined repeatedly after 3 months

Characteristics	n = 25
Age, years, mean ± SD	64 ± 12
Previous MI, n (%)	5 (20)
Previous PCI, n (%)	3 (12)
Previous CABG, n (%)	0 (0)
Diabetes mellitus, n (%)	3 (12)
Current smokers, n (%)	7 (28)
Treated with β-blocker, n (%)	6 (24)
Treated with ACE inhibitors, n (%)	5 (20)
STEMI, n (%)	13 (52)*
Anterior wall STEMI, n (%)	7 (28)*
TnT max in STEMI, ng/L	1881 [2686]
TnT max in NSTEMI, ng/L	169 [907]
Arteries stented before inclusion, n (%)	1
Stents implanted before inclusion, n (%)	2
Time from PCI to study inclusion (days)	2 [0.5]

Expressed as median [IQR] or n (%)

*MI* myocardial infarction, *PCI* percutaneous coronary intervention, *CABG* coronary artery bypass grafting, *TnT* Troponin T, *STEMI* ST-elevation myocardial infarction, *NSTEMI* non-ST-elevation myocardial infarction, *ECHO* echocardiography

**Table 2:** Echocardiographic characteristics for the included cohort, average of 2 examinations per patient

Echocardiographic variables	n = 50
Expert GLS [mean ± SD, %]	− 14.7 (± 2.4)
LVEF [% median (IQR)]	52 (4)
LVEDVI (ml/m^2^)	76 (22)
LVESVI (ml/m^2^)	37 (15)
Max LA VI (ml/m^2^)	32 (14)
Max RV AI (cm^2^/m^2^)	12 (3)
RV AF (%)	51 (13)
E/e′	10.1 (5.5)
LVMI (g/m^2^)	125 (62)
Systolic blood pressure (mmHg)	120 (15)
Heart rate (beats/min)	66 (18)

*GLS* global longitudinal strain, *RLS* regional longitudinal strain, *LV* left ventricle, *EF* ejection fraction, *EDVI* end-diastolic volume index, *ESVI* end-systolic volume index, *max LAVI* maximum left atrial volume index, *max RVAI* maximum right ventricular areal index, *RVAF* right ventricular area fraction, *E* early filling, *e*′ early diastolic tissue velocity, *LVMI* left ventricular mass index

**Table 3:** Percentage ROI widths from the three apical views as adjusted by an expert to obtain reference manually adjusted GLS and as presented by the AFI system to obtain default semi-automated GLS

ROI width	GLS Manually adjusted by an expert (n = 150)	GLS Default semi-automated (n = 150)
Narrow (%)	9	0
Medium (%)	85	15
Wide (%)	6	78
Extra wide (%)	0	7

*GLS* global longitudinal strain, *ROI* region of interest

**Table 4:** Mean default GLS, reference GLS and GLS with fixed ROIs from all 50 measurements

Default semi-automated GLS	15.3 (± 2.5)%
Manually adjusted expert GLS	− 14.7 (± 2.5)%
GLS with fixed ROI level narrow	− 15.0 (± 2.6)%
GLS with fixed ROI level medium	− 14.7 (± 2.6)%
GLS with fixed ROI level wide	− 13.5 (± 2.3)%*

Values are expressed in mean (± SD)

*GLS* global longitudinal strain, *ROI* region of interest

*p < 0.001 vs. default GLS

**Fig. 2:**
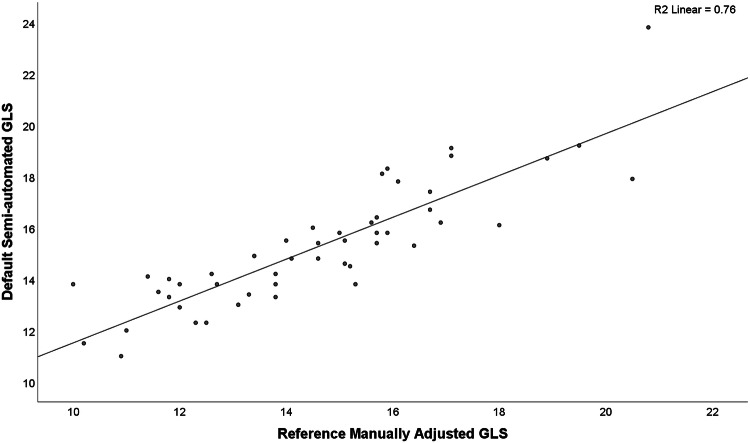
Scatterplot between the default semi-automated GLS by the vendor software and manually adjusted reference GLS by an expert with ROIs applied as presented in Table 3 Pearson’s correlation coefficient 0. 87 (p < 0.01)

Figure 2 demonstrates the excellent relationship between the default GLS and the manually adjusted expert GLS (r = 0.87, p < 0.01). The corresponding ICC was 0.93, (p < 0.001).

The present study design was based upon four different ROI patterns per examination, compliant with a total of 600 ROI analyses.

## Discussion

This study demonstrates that the difference between the default GLS by the vendor’s software and the manually adjusted GLS by the expert was not significant, and the two values were highly correlated. The only GLS value that was significantly different from those obtained from the default software assessments was with manually adjusted, but with a fixed wide ROI. As expected, the largest ROI width was associated with the lowest GLS value. These findings suggest that the default settings can be used in daily clinical echocardiographic practice and may therefore represent a desired simplification and standardization of the GLS assessment.

The feasibility of GLS measurement is strongly related to the quality of the underlying B-mode echocardiographic images. Therefore, we only selected recordings obtained by highly experienced operators and without loss of any LV segments in the three apical views. The endocardial border was well defined in all recordings, and the entire ROI, including the apical myocardium was well within the actual sector and without inclusion of the epicardium when manually adjusted by the expert. This is reflected from most manually adjusted GLS values being obtained with ROI ≤ level medium as opposed to predominantly level wide by the default GLS. Per protocol no attempts were made to manipulate ROI in default GLS analysis. All manually adjusted measurements, but with fixed ROI levels included the location of endocardium and basal parts of the LV as described, but implying that parts of the outer layer of ROI might include the epicardium, especially with the widest level. In this way we could obtain information of the influence of ROI width alone.

The patients examined represent a population where additional GLS measurements are well indicated to provide important prognostication beyond that presented by LVEF alone (1.5–7).

Although Mirea et al. recently stated that different widths of ROI significantly impact strain measurements, the literature is remarkable sparse on this topic [13]. Stoebe et al. found that GLS was significantly influenced by the ROI width in 30 healthy subjects and 15 patients with mild to moderate LV systolic dysfunction [14]. In the healthy group GLS was − 23.5% with narrow, − 20.0% with medium and − 14.6% with wide width. In those with LV dysfunction the respective values were − 12.9%, − 10.4% and − 7.6%. The percentual deviations when using the medium width as reference were 43% and 45% in the two groups respectively. Although they used the same measurements principles with a Vivid E9 machine, these differences are greater than those in our study of post AMI patients with reasonably well-preserved LV systolic function. They had used Echopac Software version 12.0.1. as opposed to the AFI system in our study. A possible explanation may therefore be that differences in ROI width levels may have been larger than in our study. The authors emphasized that standard and reference values of deformation imaging should include detailed information about the position and width of the tracking area. Spriestersbach et al. reported the influence of ROI width in 20 healthy subjects examined with a Vivid E 7 machine [15]. The Auto ROI applied was derived from a 4-chamber view only, and the endocardial border had been automatically traced based upon markers on the septal and lateral mitral ring and the apex. Initial ROI width was set by the software and two subsequent measurements were obtained from each cine loop by choosing the ROI width one step narrower and one step wider than the automatic ROI width. Mean GLS was − 21.9% with the narrower ROI, − 20.1% with the automatic and − 19.2% with the wider width. The mean percentual difference between the largest and the lowest mean GLS represented 13.4% of the automatic value, and was more within the level of our study. The authors stated that precise ROI width definition is essential in studies of the clinical impact of GLS, but added that technical factors limit its feasibility.

In view of the lack of broader scale studies on the impact of ROI, we performed a literature search on studies evaluating the prognostic role of GLS vs. LVEF for cardiac events (Supplemental file, Table 1: n = 14 studies on cardiovascular events in 8319 patients) and for LV remodeling post MI (Supplemental file, Table 2: 7 studies on 1756 patients) The majority of these studies have used manual adjustments of ROI, either in general or “when needed”. No details on ROI widths applied were provided, neither in the minority of studies with default GLS, nor in the majority with manually adjusted ROIs. In spite of all sources of technical problems listed by Mirea et al., all 21 studies were in favor of GLS as a better prognostic factor for cardiac events and LV remodeling than LVEF [13]. With those problems in mind and the possibility of publication bias, there is a need for collective efforts to standardize strain measurements as already put forward by several authors [13, 16]. Our findings, together with the two aforementioned studies emphasize that such recommendations should also include the proper use of manually adjusted versus default GLS values.

The apparent paradox that default GLS with mainly wide ROIs gave similar values that were obtained with manually adjusted ROIs narrow and medium may be explained as follows: The automatic ROI presented is usually closer to the endocardium/LV cavity compared to manually adjusted ROI and will thereby avoid epicardium even with wide and extra wide ROIs (which will take down the strain estimate). The main reason for the differences observed, however, is probably related to the apical region where the automatically presented ROIs sometimes are too much into the LV cavity. This is caused by lower image quality in the apex due to near field noise in the image. Both in clinical practice and according to the vendors experience it is quite common that the user has to move the ROI further out in the apical region, as also applied in the present study. This will inevitably reduce GLS values from adjusted ROIs with the wide level that is prone to affect the epicardium more than with the two more narrow levels.

The different location of default versus manually adjusted ROIs may seem distrustful from anatomical considerations. Strain measurements should in principal not be based on speckle tracking within blood, but preferably in the myocardium. Also, inclusion of “pseudo contraction” of basal left atrial walls the aortic root in several cases should, to our opinion, be avoided. On the other hand, adjustments of ROI according to the Negishi guidance [10], as performed in our study may be considered arbitrary and not necessarily correct.

Our findings did not, however, reveal any major problems with the default assessment by the automated software. This may result in making the speckle tracking more widespread among users who are skeptical to comply with technical challenges and time-consuming aspects associated with editing.

To elucidate problems with variations of ROI for GLS values reported, a prerequisite for future studies would be more detailed information on adjusted ROI location and width and the contribution of default GLS measurements applied. Such studies may also form the basis for guidelines which may encourage clinicians to include GLS measurements in their clinical practice. So far, until guidelines and new vendor developments occur, we believe that the use of edited ROI can be applied in keeping with the Negishi guidance (8), but with the modification that wider widths (≥ “medium”) should be avoided. As evident from the present study, however, there is no apparent major error to simply use default GLS provided that the grey scale apical recordings are of excellent quality.

### Study limitations

Like in most studies on the prognostic impact of GLS, the results are derived from GE Vingmed equipment, and are therefore limited to these machines and software. The initiation of the EACVI/ASE Strain Standardization task force and a consensus document for a standard of myocardial deformation imaging have made variability between different machine and software vendors lower in GLS than in other conventional echocardiographic indices of LV function [18]. Because our study focused on methodological aspects only, the examinations included were from highly selected echogenic patients, and our results may not be applicable for general practice. With such a limited population and, more importantly, the relatively small variability of GLS our results are not conclusive for a more general population with various aspects of cardiac disease. Our results may therefore not apply to patients with larger contraction abnormalities where possibly a wide ROI can be useful or a narrow ROI insufficient, or with reduced GLS. With these reservations in mind reliable results were strongly related to the quality of the apical grey-scale views obtained, since a number of segments may otherwise need to be excluded from the analysis due to poor image quality or tracking. For fixed default GLS, deviation of ROI in poor quality recordings may render our results to be unrealistic for a general post MI population. These technical considerations clearly represent a limitation of general application of default GLS and thus represent a disadvantage versus the more robust measurements of LVEF and volumes from unselected recordings. An unavoidable problem is that all adjusted measurements were based upon the default ROIs presented in each view. Such a bias may have influence on the apparent similarity obtained with adjusted values with the narrow and medium ROI levels. In order to avoid such bias, all adjustments were performed in a similar manner regardless of the actual ROI levels selected. Finally, these results were based on a relatively small cohort of patients with PCI treated acute MI and may not be representative for patients with different cardiovascular diseases. A further selection bias was the use of two examinations per patients included instead of one per 50 different patients. With the present design, however, our images have been selected from highly echogenic patients leaving the role of different ROIs to be the predominant factor for GLS differences. Although the study sample was relatively small, the present results are based upon a large number of ROI analyses.

## Conclusions

The difference between the default GLS values from the vendor software and the GLS values manually adjusted by an expert were equal. However, when choosing the widest ROI width by manual adjustment the results were significantly different from default and expert measures. With manually adjusted narrow and medium ROIs the differences versus default GLS were neglectable. Our results may infer less need for adjustments in clinical practice and future studies, but these interpretations are limited to the software and hardware tested in our study.

## Electronic supplementary material

Below is the link to the electronic supplementary material.

Supplementary file1 (DOCX 20 kb)
