# Supplementary material for: The impact of semi-automatic versus manually adjusted assessment of global longitudinal strain in post-myocardial infarction patients
Source: Int J Cardiovasc Imaging. 2020 Mar 31;36(7):1283–90. doi: 10.1007/s10554-020-01826-4 (PMC7256100; doi:10.1007/s10554-020-01826-4)
Supplement: Supplementary file 1 — Supplementary file1 (DOCX 20 kb) [file 10554_2020_1826_MOESM1_ESM.docx]

**Supplemental file 1, table 1, Studies on GLS as predictor of clinical events, mostly compared with LVEF as predictor, all based upon apical GLS triplane recordings and a minimum of 60 patients included**

Study, year n patients Vendor Editing ROI level info GLS Positive for EP

Stanton 2009 (1) 546 consecutive for echo GE 7 manually adjusted if failed segment No Yes, TM, > LVEF

Mignot 2010 (2) 145 HF LVEF≤ 45% GE 7 Adequate tracking by adjusting ROI No YES, MACE >LV EF

Sjøli 2011 (3) 77 Post Tr.ly. for STEMI GE 7 n.a. No Yes, MACE, > LVEF

Munk 2012 (4) 576 Post PCI for STEMI GE 7 ROI manually adjusted No Yes, MACE > LVEF

Bertini 2012 (5) 1060 Chronic isch. CMP GE 7 ROI manually adjusted No Related to TM and HF hosp.

Ersbøll 2013 (6) 849 Post AM - LVEF > 40% GE 9 ROI adjusted, when needed No Related to CVM and HF hosp.

Ersbøll 2013 (7) 988 Post AMI GE 9 Manual correction , if needed No Related to SD and mal. VA

Su 2013 (8) 196 Persistent AF GE 7 ROI manually adjusted Min. 8 mm Yes, MACE > than LV EF

Haugaa 2013 (9) 569 > 40 days post MI GE 7+9 n.a. No YES, + MD for VT and SD

Stampehl 2014 (10) 450 HF with EF< and ≥ 50% GE 7 n.a. No Yes, to CVM and rehosp >LVEF

Saito 2015 (11) 468 First echo for HF GE 7+9 Automatic tracking of ROI only No Yes, > LVEF for readm

Sengeløv 2015 (12) 1065 Adm. HF- LVEF< 45% GE 7+9 ROI edited, if necessary No Yes, for TM > LVEF

Choi 2015 (13) 637 Post AMI n.a. n.a. n.a. Yes, for MACE > than LVEF

Cha 2017 (14) 691 Post PCI for STEMI GE Echopac BT 12 n.a. n.a. Yes, MACE > than LVEF

Abbreviations: GE 7 = Vivid 7/9 echo machine. ROI = region of interest; TM = Total mortality; HF = heart failure; MACE = major adverse cardiovascular Events; Tr. Ly. = thromboplysis; isch = ischemic; CMP = cardiomyopathy; Hosp. = hospitalization; CVM = cardiovascular mortality; SD = Sudden death; mal. = Malignant; VA = ventricular arrhythmia; MD = Mechanical Dispersion. VT = ventricular tachycardia; Readm. = readmission to hospital; Adm. = admitted

**Supplemetal file 1, table 2. GLS as predictor for LV remodelling , defined as an increase of left ventricular end-diastolic or end-systolic volume indexes LVEDVI or LVESVI) at 3-9 months following AMI. All studies are based upon apical triplane measurements of GLS and a minimum of 60 patients included**

**Study, year n patients Vendor Editing ROI lev. info GLS predictor for remodeling (LVr)**

***Definition > 15% increase of LV ESVI***

Altiok 2014 (15) 93 PCI treated AMI GE 7 manual correction applicable No Yes, comparable with LGE CMR

Cong 2015 (16) 127 PCI treated STEMI GE 7 manual editing, if necessary No For LVr and AE

***Definition > 20% increase of LVEDVI***

Bochenek 2011 (17) 66 PCI treated STEMI GE 7 manual corrections entered No Yes

Zaliaduonite-Peksiene (18) 141 PCI treated STEMI GE 7 n.a. No May predict

Joyce 2014 (19) 1041 PCI treated STEMI GE7 manual adjustment to M thickness No Independent association with LVr

Lacalzada 2015 (20) 97 PCI treated STEMI Ph ie33 automatic adjustment to equal M th. No Excellent

Lu 2017 (21) 110 PCI treated STEMI GE 9 manual tracing of endocardial border No Independent

Hu 2017 (22) 81 Post AMI patients GEV 9 n.a. Yes§ Valuable

§ ROI selected by wall motion score index scores ≥ 2

Abbreviation as in table 1, otherwise: : LGE CMR = late gadolium enhancement cardiac magnetic resonance; AE = Adverse events; M = Myocardium; th = thickness; Ph = Philips

**References to the studies in tables 1 and 2**

1. Stanton T,Leano R,Marwick TH. Prediction of all-cause mortality from global longitudinal speckle strain,. Circ Cardiovasc Imaging 2009; 2: 356-64.
2. Mignot A, Duval E, Zaroui A et al. Global longitudinal strain as a major predictor of cardiac events in patients with depressed left ventricular function: a multicenter study. J Am Soc Echocardiogr 2010; 23: 1019-24.
3. Sjøli B, Grenne B, Smiseth OA, Edvardsen T, Brunvand H. The advantage of global longitudinal strain compared to left ventricular ejection fraction to predict outcome after myocardial infarction. Echocardiography 2011; 28: 556-63.
4. Munk K, Andersen NH, Terkelsen CJ et al. Global left ventricular longitudinal systolic strain for early risk assessment in patients with acute myocardial infarction treated with primary percutaneous intervention. J Am Soc Echocardiography 2012; 25: 644-51.
5. Bertini M, Arnold CT, Antoni ML et al. Global longitudinal strain predicts long-term survival in patients with chronic ischemic cardiomyopathy. Circ Cardiovasc Imaging 2012; 383.91.
6. Ersbøll M, Valeur N, Mogensen EM et al. Prediction of all-cause mortality and heart failure admissions from global longitudinal strain in patients with acute myocardial infarction and preserved left ventricular ejection fraction. J Am Coll Cardiol 2+13; 61: 2365-73.
7. Ersbøll M, Valeur N, Andersen LJ et al. Early echocardiographic deformation analysis for the prediction of sudden cardiac death and life-threatening arrhythmias after myocardial infarction. J Am Coll Cardiol Img 2013; 6: 851-60.
8. Su H-M, Lin T-H, Hsu P-C et al. Global left ventricular systolic strain as a major predictor of cardiovascular events in patients with atrial fibrillation. Hear 2013; 99: 1588-96.
9. Haugaa K, Glenne BJ, Eek CH et al. Strain echocardiography improves risk prediction ov ventricular arrhythmias after myocardial infarction. J Am Coll Img 2013; 6: 841.
10. Stampehl MR, Mann DJ, Nguyen JS, Cota F, Colmenares C, Dokainishi H. Speckle strain echocardiography predicts outcome in patients with heart failure with both depressed and preserved left ventricular ejection fraction. Echocardiographyt2015; 32: 71-8.
11. Saiti M, Negishi K, Eskandari M. Association of left ventricular strain with 30-day mortality and readmission in patients with heart failure, J Am Soc Echocardiogr 2015; 28: 652-6.
12. Sengeløv M, Gofsk Jørgensen P, Skov Jensen J et al. Global longitudinal strain is a superior predictor of all-cause mortality in heart failure with reduced ejection fraction. J Am Coll Cardiol Img 2015; 1351-9.
13. Choi AW, Park J-H, Sun BJ et al. Impaired two-dimensional global longitudinal strain of left ventricle predicts adverse long-term clinicalk outcomes in patients with acute myocardial infarction. In J Cardiol 2015; 196: 165-7
14. Cha M-J, Kim H-S, Kim S H, Park J-H, Cho G-Y. Prognostic power of global 2D strain according to left ventricular ejection fraction in patients with ST elevation myocardial infarction. PLOS ONE 12 (3): e0174160,https://doi.org/10.137/journal. Phone 0174160
15. Altiok E, Tienmann S, Becker M et al. Myocardial deformation imaging by two-dimensional speckle tracking echocardiography for prediction of global and segmental functional changes after acute myocardial infarction: A comparison with late gadolium enhancement cardiac magnetic resonance. J Am Soc Echocardiogr 2014; 27: 249-57.
16. Cong T, Sun Y, Shang Z et al. Prognostic value of speckle tracking echocardiography in patients with ST-elevation myocardial infarction treated with late percutaneous intervention. Echocardiography 2015; 32: 1384-91.
17. Bochenek T, Wita K, Tabor Z et al. Value of speckle-tracking echocardiography for prediction of left ventricular remodeling in patients with ST-elevation myocardial infarction treated by primary percutaneous intervention. Echocardiogr 2011; 24: 1342-8.
18. Zaliaduonyte-Peksiene D, Simonyte S. Lesauskaite V et al. Left ventricular remodeling after acute myocardial infarction: Impact of clinical, echocardiographic parameters and polymorphism of angiotensin gene. Journal of Renin-Angiotensin-Aldosterone System 2014; 15: 286-93.
19. Joyce E, Hoogslag GE, Leong DP et al. Association between left ventricular global longitudinal strain and adverse left ventricular dilatation after ST-segment- elevation myocardial infarction. Circ Cardiovasc Imaging 2014; 7: 74-81
20. Lacalzada J, de la Rosa A, Izquierdo MM et al. Left ventricular global longitudinal systolic strain predicts remodeling and subsequent events in patients with acute myocardial infarction treated with primary percutaneous intervention. Int J Cardiovasc Imaging 2015; 31: 575-84.
21. Xu L, Huang X, Ma J et al. Value of three-dimensional strain parameters for predicting left ventricular remodeling after ST-elevation myocardial infarction. Int J Cardiovasc Imaging 2017; 33: 663-73.
22. Hu B, Zhou Q, Chen J et al. Prediction for improvement and remodeling in first-onset myocardial infarction by speckle tracking echocardiography: is global or regional strain better? Ultrasound in Med & Biol. “017; 43: 2452-60.
